# Causes of stigma and discrimination associated with tuberculosis in Nepal: a qualitative study

**DOI:** 10.1186/1471-2458-7-211

**Published:** 2007-08-16

**Authors:** Sushil C Baral, Deepak K Karki, James N Newell

**Affiliations:** 1 Nuffield Centre for International Health and Development, Leeds Institute for Health Sciences, University of Leeds, Charles Thackrah Building, 101 Clarendon Road, Leeds, LS2 9LJ, UK; 2 Health Research and Social Development Forum (HERD), PO Box 24133, Kathmandu, Nepal; 3 National Centre for AIDS and STD Control (NCASC), Ministry of Health and Population (MoHP), Kathmandu, Nepal

## Abstract

**Background:**

Tuberculosis (TB) is a major cause of death. The condition is highly stigmatised, with considerable discrimination towards sufferers. Although there have been several studies assessing the extent of such discrimination, there is little published research explicitly investigating the causes of the stigma and discrimination associated with TB. The objectives of our research were therefore to take the first steps towards determining the causes of discrimination associated with TB.

**Methods:**

Data collection was performed in Kathmandu, Nepal. Thirty four in-depth interviews were performed with TB patients, family members of patients, and members of the community.

**Results:**

Causes of self-discrimination identified included fear of transmitting TB, and avoiding gossip and potential discrimination. Causes of discrimination by members of the general public included: fear of a perceived risk of infection; perceived links between TB and other causes of discrimination, particularly poverty and low caste; perceived links between TB and disreputable behaviour; and perceptions that TB was a divine punishment. Furthermore, some patients felt they were discriminated against by health workers

**Conclusion:**

A comprehensive package of interventions, tailored to the local context, will be needed to address the multiple causes of discrimination identified: basic population-wide health education is unlikely to be effective.

## Background

Tuberculosis (TB) is a leading cause of adult death in the world, killing 1.7 million people each year. Globally, 14.6 million people have active TB disease: each year 8.9 million people develop active TB [1]. The impact of TB is such that in 1993 WHO declared TB to be a global emergency.

TB is closely associated with poverty: although all ranks of society get the disease, the poor are at greatest risk, both because they are in greater contact with other sufferers (because of overcrowding at home, at work, travelling and socialising), and because their immune system is weakened due to poor nutrition [2]. A major impact of TB on marginalised people with little social capital is to push them into abject poverty, because it destroys their ability to earn money or subsist through their work, and because the diagnostic and treatment processes are often costly.

TB is transmitted by droplets in the air produced when an infected person coughs, and inhaled by surrounding people [3]. The only effective approach to TB control is to rapidly diagnose and treat TB patients, thereby breaking the chain of infection. Although treatment renders infected people non-infectious within two weeks of commencement [4], TB is not cured until completion of a full course of treatment lasting 6–8 months which requires regular visits to a health centre (daily for the first two months; daily or weekly thereafter). Both these visits to health centres and the symptoms of TB (chronic cough, weight loss and weakness) make it difficult for sufferers to disguise the fact that they have TB. Once identified, sufferers experience considerable stigma and discrimination on account of their disease, leading to delays to diagnosis and treatment and causing a major impact on TB control [5].

### Stigma and discrimination

The words stigma and discrimination are often used interchangeably, and this can lead to confusion.

Stigma is defined by Goffman [6], the seminal author on stigma, as an 'attribute that is deeply discrediting' and that reduces the bearer from 'a whole and usual person to a tainted, discounted one'. In Goffman's view, stigma commonly results from a transformation of the body, blemish of the individual character, or membership of a despised group. He emphasizes the relationship between an attribute and a stereotype. Building upon this definition, Link and Phelan [7] define stigma as 'stigma exists when a person is identified by a label that sets the person apart and links the person to undesirable stereotypes that result in unfair treatment and discrimination'.

Discrimination occurs in two forms. Direct discrimination occurs when a person is treated less favourably, on the grounds of their disease, than others are or would be treated in the same or similar circumstances. Indirect discrimination occurs when a requirement or condition is applied which, although applied equally to all persons, is such that a considerably smaller proportion of people with the disease can comply with it and it cannot be shown to be justifiable other than on health grounds. (These definitions paraphrase the definitions of discrimination in UK Acts of Parliament outlawing discrimination on grounds of sex [8], race [9] and disability [10].)

It is thus the perception of stigma by other people or by the stigmatised person him/herself that leads to discrimination: and therefore stigma is important because of the way it causes discrimination. Much such discrimination may be self-discrimination [11], where an individual feels unworthy or guilty, leading to a lack of self-worth and depression and abnormal behaviour such as self-isolation, avoidance behaviour and introversion [12].

Many communicable diseases, such as TB [13], HIV/AIDS [14] and leprosy [15], are associated with stigma and discrimination. Stigma and discrimination have an enormous impact on sufferers [16]. That impact is felt at home, in the workplace and institutions [17] and in the community. The obvious cause of discrimination is a fear of being infected, yet stigma and discrimination also occur for non-infectious diseases such as cancers [18] and mental health problems [19]. Stigma associated with many diseases is a result of prejudice, which can be split into two categories: instrumental and symbolic attitudes [20]. The former refers to the material advantages and disadvantages caused by the relationship between the discriminator and the sufferer – an example is (perceived) risk of infection – while the latter refers to the gains and losses in the process of reaffirming personal values that are produced by the interaction between the discriminator and the sufferer – an example is concern about being labelled as 'poor' by association. Some of the multiple causes are conjectured to be fear of infection or pollution, fear of the different, association of the condition with poverty or other discrediting factors, and a means of upholding existing power differentials. There may be considerable ambivalence in the minds of health workers and family members as they attempt to balance desires to help sufferers while still retaining prejudices and behaving in discriminatory ways [21]. The impact on sufferers is considerable, yet there is little understanding of the precise nature of the causes of stigma and discrimination.

### The impact of stigma and discrimination on TB care and control

Many authors [22-27] describe the effects of stigma and discrimination associated with TB. The principal effects in developing countries are social isolation of patients, both outside the family, where the person may be avoided by former friends and acquaintances, and inside the family where the patient may be forced to eat and sleep separately [28,29]. Patients often isolate themselves to avoid infecting others and to avoid uncomfortable situations such as being shunned or becoming the subject of gossip. Being either a patient or an ex-patient is likely to affect employment and employment prospects. Unmarried women often find it difficult to get married, due to discrimination by prospective husbands and in-laws, while married women may find they are divorced because they have TB or if a history of TB is subsequently revealed.

Stigma and consequent discrimination have a double impact on TB control. First, concerns about being identified as a person with TB make it more difficult for people with a cough of long duration who suspect they may have TB to seek care, because of the public nature of the TB diagnostic process. By delaying seeking care, these people may develop more serious symptoms, meaning they will be more difficult to treat; and as they remain infectious for longer, they are more likely to transmit the disease to others. Second, concerns about stigma and discrimination for TB make it more difficult for patients to continue with care, because their fears of being identified as being, or having been infected with TB hinder their access to services on a daily basis. Again, this can lead to serious symptoms and increased transmission.

Despite the importance of stigma and discrimination in restricting sufferers' access to diagnosis and treatment, the international strategy for TB control published by StopTB (the WHO agency with responsibility for TB control) has until the latest revision [30] in 2006 treated TB as a biomedical rather than a social disease, and showed little awareness of the need for patient-centred approaches. Even in this 2006 revision, there is no mention of stigma and discrimination associated with TB, even though publications by the same organisation repeatedly acknowledge that TB is a highly stigmatised disease [1].

### Causes of stigma and discrimination associated with TB

There are few published works we are aware of that attempt to determine the causes (rather than the existence or effects) of stigma and discrimination associated with TB. One addresses social stigma associated with TB in Nicaragua [31]. This study described two pairs of contradictory influences on stigma and discrimination: (a) feelings of affection and supportive attitudes towards people affected by TB, countered by the fear of transmission of TB; and (b) sympathy for people affected by TB considered to be unlucky, contrasted with mistrust of people affected by TB considered to have brought the disease upon themselves. This leads to self-stigmatisation and discrimination. Issues of power and knowledge further mediate the situation. Another study deals with a very specific group of potential discriminators: nurse instructors in various (unspecified) countries [32]. The main findings were that the main causes were fear of contracting the disease (58%), association with poverty (40%) and lack of knowledge (34%). The percentages quoted come from a self-selected sample of respondents, and are therefore not representative of any specific population group, so care must be taken in interpreting these results. Other studies not focusing specifically on stigma and discrimination identify the following determinants of stigma and discrimination associated with TB: unfounded beliefs about transmission [23,33,34]; health staff attitudes [35]; and associations with other potential sources of discrimination [36].

Without knowledge of the causes of stigma and discrimination associated with TB, it is difficult to devise discrimination-reduction strategies that are likely to work. There is therefore a need to investigate the causes of discrimination in depth from patients' and families' perspectives. We therefore decided to investigate stigma and discrimination associated with TB in Nepal, where TB is a major cause of morbidity and mortality, and issues related to stigma and discrimination provide substantial barriers to care-seeking and treatment completion. It is already well established that discrimination takes place as a result of TB, and there have been many reports on the forms that that discrimination takes. The objectives of our research were to build on this knowledge by taking the first steps towards determining the causes of discrimination associated with TB, which will then inform possible interventions to reduce such discrimination.

## Methods

Data collection was performed in the Kathmandu valley, Nepal. In-depth interviews were performed with TB patients, family members of patients, and members of the community. All interviews were performed in Nepali or Newari by native speakers. Tapes of the interviews were translated into English and initial themes identified by the interviewer.

The focus of the interviews was causes rather than descriptions of discrimination. In the first stage of the interview, interviewees determined themes rather than being guided by the interviewer. In the second stage, interviewees were prompted on any of the themes that had not already been covered. Interviewing and analysis followed the grounded theory approach. Themes were identified based on their recurrence and/or wider relevance.

Care was taken to avoid confusing the order and apparent ease of recall of issues with their importance to the interviewee. Due to the sensitive nature of the interviews, issues that interviewees initially identify, might only have had superficial importance, and issues which were disclosed subsequently, might have been of major importance, but more painful and therefore harder to disclose.

Interviewers were chosen for their ability to listen to empathise with interviewees. Interviewers were generally of the same sex as interviewees. Where necessary, interviewees were interviewed on more than one occasion, to build trust and rapport, and to probe specific issues.

Informed consent was obtained from all interviewees, and ethical approval was granted by the Board of the Nepal Health Research Council.

## Results

We interviewed:

• 21 TB patients: 5 young male, 7 young female, 6 older male, 3 older female. A range of castes was included.

• 5 family members of TB patients.

• 8 members of the community who were not current TB patients or family members of TB patients.

Interviews took on average about 2 hours, and many interviewees were subsequently re-interviewed to clarify issues. No-one identified as a potential interviewee refused to be interviewed, although several interviewees asked that the interview take place in a discrete location to avoid being seen by anyone known to the interviewee.

### Existence and causes of self-stigma and -discrimination

It was clear that patients' beliefs were a major cause of self-discrimination. TB patients generally isolated themselves from family and friends, and particularly from children, because of a fear of transmitting the disease.

"I was afraid of TB because I could transmit that to my son, daughter and children. I am old now, and it will not make that much problem if I die but if my son, daughter and children die then it will be a problem. I thought in such a way and was afraid of this disease."

(53 year old female patient, unemployed, code 17.)

This behaviour continued throughout treatment, even among patients who were clearly advised that they posed no risk to their family.

"My husband knows that I am suffering from TB. He had taken me for check-up. He told me that whatever the doctor said, I was suffering from TB and should not give jutho [eat food from the same plate] to children because it is danger and communicable disease. We also should not sleep together in a single bed."

(32 year old female patient, vegetable seller, code 21.)

TB patients isolated themselves from friends and family, not only because of a fear of infecting others, but because of fear of discrimination.

"I felt odd to sit together with friends. Friends said nothing but I myself felt so while taking foods etc. Though I had idea that it is a curable disease, I felt whether I would live no longer or it would happen for ever, for whole life time. ...If my friends open my bag and there is my treatment card, I become afraid, thinking what they could have thought, and feeling fear of whether they change their behaviour towards me, like not sitting with me, not taking food together, and not talking in a good way."

(18 year old female patient, university student, code 05.)

*If someone special is there to whom I must say then I say that I am suffering from TB, otherwise why say to everyone? If they know they may say he is sick and suffering from TB. Other people may hate saying that I am suffering from TB*.

(41 year old male patient, tailor, code 14.)

"Nobody in my family knows about my disease. My wife also does not know about my disease. I am taking drugs regularly and I have not told her. I think they may be stressed [by the knowledge]. ... If I say in my home it will only create tension in other family members. ... If my wife knows that I am sick then I think she would be tense and feel heavy mental burden because I am the sole bread earner for my family."

(37 year old male patient, code 20.)

This was not always the case, however:

"There are two types of people: one who suffers from this disease and knows that it will be cured after taking drugs; and another, who afraid of thinking that others may know about their sufferings. I don't know why they think so and become so afraid. But so far as I think, one should not think in such a way."

(18 year old female patient, school student, code 06.)

Fear of gossip was a recurrent explanation for hiding TB from friends and neighbours:

"Thinking that neighbours might hate [him] I had not told them about his disease. ... We went to the village after his diagnosis, but I didn't say that he is suffering from such disease because it was village and once they know they start back-biting and may hate us."

(Mother of young male TB patient, housewife, code 16a.)

"None of my friends know that I am suffering from TB. I didn't let them know. I don't have faith in friends so I don't say with friends. They do back-biting and if they get a small issue they make it larger and so I didn't share this suffering with them."

(Young female patient, code 22.)

These concerns about discrimination by others may come from historical hangovers from the days when TB was much more difficult to cure, coupled with limited understanding in village communities that things have changed:

"This stigma in TB is already been socialised in our society since a long time."

(34 year old male patient, university lecturer, code 03.)

"Although they usually hate TB sufferers, they have said nothing to me."

(16 year old female patient, school student, code 10.)

"Nowadays it is not like that of older times; community [ie village] people now take it [TB] as a normal thing."

(53 year old female patient, unemployed, code 17.)

Concerns about the incurability of TB continue, even though there is widespread first-hand experience of friends or neighbours being fully cured following a diagnosis of TB. This was the case more often than not with our respondents:

"One lady of my age had also suffered from TB in my neighbourhood and other friends were also suffering from it. So, I had already heard about this disease before I suffered."

(19 year old female patient, factory worker, code 11.)

"My cousin had also suffered from TB. So I thought of it as a normal disease."

(Male patient, school student, code 16.)

Unmarried TB patients do not report being overly worried about the impact of TB on their marriage prospects. One respondent reported:

"My husband, his mother and uncle knew about my disease before marriage. When they came to propose ... we had said that I was suffering from TB and taking drugs and they said it can be treated. ... We had arranged marriage. I had seen him only once before marriage."

(19 year old female patient, factory worker, code 11.)

On the same topic, another respondent stated his belief that

"I don't think any girl will refuse to marry me just because of my suffering from TB."

(25 year old male patient, cook, code 15.)

However, in Nepal issues of marriage are highly confounded with gender issues. One respondent commented

"Even if you are not TB patient they treat you differently [ie badly] because you are girl."

(18 year old female patient, student, code 19.)

### Existence and causes of stigma and discrimination by family and friends

We found little evidence of discrimination within the family:

"Other family members also know that she is suffering from TB and said nothing. Everyone in her maternal home and relatives know but nobody hates her. They just suggested that it will be cured: she needs to eat in a good way and not neglect herself. Neighbours also know and do not hate. They also frequently visit and sit together. We are only four in the family and neighbours help in bringing food and in other things. Friends also helped a lot. They bought drugs at night time also."

(Mother of young female TB patient, manufacturer of temple gods, code 13.)

There was also little evidence that in reality friends would discriminate against a TB patient. On the contrary, respondents reported their friends provided encouragement and support.

"There are three to four friends [who I work with]. They know about my suffering. They said 'I know you are suffering from such disease – tell us. We can also bring drugs for you.' They say 'Don't hide your disease, tell us. We will help you as much as possible."'

(43 year old male patient, labourer, code 08.)

"Everyone in my birth-home knows about my disease. Everyone knows about my suffering and so I am taking drugs. They didn't feel anything wrong. All of them love and don't hate me – rather they take care more."

(19 year old female patient, factory worker, code 11.)

When it was suggested that friends might change their attitude to a person after discovering he had TB, the response was

"I have said to every close friend ... that I am suffering from TB. They also suggested I should not worry: 'It will be cured by taking drugs'. There is no such change in behaviour of my friends."

(Unemployed male patient, code 09.)

Likewise we found little evidence of discrimination by neighbours:

"Neighbours nearby my home and members of my [workplace] know about my suffering. They act normally. Their behaviour is not changed by merely knowing that I am suffering from such disease."

(18 year old female patient, university student, code 05.)

"House owner and people in nearby rooms and area also know about my disease. They say nothing. Some of the women had also suffered from TB and got cured. I sit and eat together with my neighbours but I have felt nothing uneasy of any difficulty."

(19 year old female patient, factory worker, code 11.)

### Existence and causes of institutional stigma and discrimination

Some patients felt they were discriminated against by health workers.

"... one nurse, who seemed quite mature, looked like very much fearing that it [TB] could be transmitted to her. She suggested to me to put on the mask every time and not to put it off even for a fraction of time as if she was alarmed and saying to me 'be careful."'

(18 year old female patient, code 06.)

"I'll tell you what happened to me when I was suspected of TB. ... [At his first visit to a hospital while seeking diagnosis] I mentioned that I saw blood in my sputum last week and came here for treatment. Suddenly and surprisingly she asked me to stand far from the window and she covered her mouth, her behaviour was changed. I got surprised and worried I may have got a dangerous disease. I felt so scared and [went] to the room [ie home] without any check up. ...Now I do not want to remember that time."

(29 year old community member, code 26.)

Others reported more positive experiences, and were happy with the way staff behaved towards them. We have not yet carried out studies to identify the causes of this discrimination by health workers. Anecdotally and from observation of health workers' behaviour towards patients, it is clear that some health workers are concerned about the risk of becoming infected with TB, but further research is needed.

Respondents who were students, either at school or university, reported they had not experienced and did not fear discrimination from teachers. However, students tended not to tell their teachers.

"Teachers do not know that I am suffering from such disease. Even if they did know, nothing would have happened."

(16 year old female patient, school student, code 10.)

"Teachers might not know about my disease. They don't ask and so I haven't said. Only three to four of them know it. ... [a specific] teacher is friendly and asked me and I said I am suffering from TB."

(Male patient (age unspecified), school student, code 16.)

### Existence and causes of stigma and discrimination in the community

Most community members interviewed commented that people with TB are easily identifiable because of physical weakness, thinness and cough: one gave a list of physical signs, and also remarked that

"these people usually do not like to talk and come around other people unless [there is a] specific reason."

(59 year old male community member, retired, code C01.)

Although patients and family members expressed their confidence that community members did not discriminate against them, one community member interviewed stated that in fact she did discriminate.

"As the disease can transmit to other people, there is risk of getting disease in the community. So, we need to take care of ourselves. How? – we should not meet with the people who have TB; we should not visit the homes of those who do have any of the members got TB."

(55 year old female community member, office assistant, code C02.)

One respondent made an interesting comparison between TB and typhoid (another communicable disease):

"A patient infected with typhoid, people take care of disease and carry them to hospital for treatment but they do not behave equal with TB patients."

(34 year old male professional, code C07)

TB was associated with what is considered in Nepal to be immoral behaviour (visiting prostitutes, drinking and smoking):

"When a woman got TB she was looked at with the eyes of suspicion whether she would have gone for sex with low profile people [ie she was suspected of being promiscuous]. This kind of feeling is still there in the community even today."

(59 year old male community member, retired, code C01.)

"In the community TB can easily be guessed to those people who do have a habit of drinking alcohol and smoking cigarettes. People seem quite thin and usually not interest for food."

(55 year old female community member, office assistant, code C02.)

"We have seen and heard that TB usually got to those who drinks, who smokes, who [goes to many] prostitutes, who drives trucks, who do not care of food [ie who eat a lot]."

(69 year old retired headmaster, code C04.)

Furthermore, many respondents believed that TB was associated with other 'disreputable' attributes: being poor, and of low caste. One respondent (a 65 year old housewife, code C05) also commented that TB was associated with working most of the time in the fields, and with a lack of adherence to religious rituals.

One respondent (a 34 year old male professional, code C07) believed that TB was not only communicable, but also a hereditary disease.

Several community members commented that women who had TB would be discriminated against.

*People do scare to get marry with the lady with TB, even after she got cured because it is believed that the lady who experienced TB in her life would not beget any children. She would never be fertile*.

(59 year old male community member, retired, code C01.)

The comment by patient 11 quoted above was contradicted by her husband, who independently said

"If I had known she was suffering from TB I would not have married her. But I knew after marriage only. Now I have accepted it taking it as my fate. I advise her not to be afraid and not to worry about it."

(Unemployed husband of female patient, factory worker, code 11a.)

For reasons of confidentiality we were unable to clarify this situation. Despite probing, the husband did not elaborate why if he had known his wife-to-be was suffering from TB he would not have married her.

The patient clarified that

"He might have said that he had no idea beforehand, might be because of odd feeling in saying so in front of you."

(19 year old female patient, factory worker, code 11.)

One respondent stated

*"... if a girl gets TB, community people will be reluctant to arrange marriage with her, because TB is also perceived in community that it is a divine curse to the person and family, comes from wrong things they did in the past. This belief is so strong in the community ..."*.

(34 year old male professional, code C07)

### Other concerns

Several patients reported they were relieved to find they had TB rather than cancer: they knew that TB was curable, whereas they believed cancer was not – probably rightly for poor people in Nepal.

"I worried much that it might be cancer."

(Mother of young female TB patient, manufacturer of temple gods, code 13.)

"We had suspected it as a cancer.... If it was cancer then what could we poor people do?"

(Mother of young male TB patient, housewife, code 16a.)

"When it was diagnosed first, I was suspecting it as a cancer."

(37 year old male patient, code 20.)

## Discussion

### Summary of main findings

Most respondents with TB reported that they isolated themselves from family and friends, partly to avoid infecting them. This self-discrimination remained high throughout the eight months of treatment. Another reason respondents isolated themselves from friends was to avoid gossip and potential discrimination. Patients also hid their TB from other members of the community. Furthermore, some patients felt they were discriminated against by health workers, and were able to quote specific examples of discrimination.

We identified multiple causes of discrimination against people with TB by individual members of the community unconnected with the patient (ie not family or friends). These included

• fear of a perceived risk of infection

• perceived links between TB and other causes of discrimination, particularly poverty and low caste

• perceived links between TB and disreputable behaviour, particularly drinking alcohol, smoking tobacco and visiting sex-workers

• perceptions that TB was a divine curse sent down to punish former unacceptable behaviour.

### Analysis and implications for addressing TB-related discrimination in Nepal

There appears to be a mismatch between TB patients' fears of negative responses by friends to a disclosure of TB, and the reality of the encouragement and support provided by friends towards patients who actually disclosed their infection. This mismatch provides a potential avenue for interventions to reduce discrimination associated with TB. If patients are made aware of the support that is being provided to other TB patients by friends and neighbours, they may be more open about their disease, with two potential benefits: psychologically, they may gain from not having to hide their disease; and materially, they may gain encouragement and support themselves. However, before implementing this strategy widely, it will be necessary first to confirm the cause-effect mechanism involved. If disclosure causes supportive behaviour from others, interventions to encourage disclosure may be useful; but if the mechanism is actually that having supportive friends and neighbours facilitates disclosure, such interventions may prove harmful. Even if the former is the case, it would be wise to test such a strategy on a small scale before expansion, to ensure that unexpected consequences do not arise.

Although patients are routinely told in the Nepali health system that TB stops being infectious at most two weeks after commencement of treatment, self-discrimination due to TB remained high throughout the eight months of treatment. Better health education or effective health counselling for patients might help to allay these fears: although with increasing rates of multi-drug resistant (MDR) TB, and now extremely drug resistant (XDR) TB, perhaps patients' fears are becoming more justified. Global practice differs as to whether such people should be isolated to protect others – in industrialised countries they generally are, while isolation is rare in developing countries. There does not seem to be consensus whether discrimination is acceptable under such circumstances. In the case of TB sufferers who are untreated or who recently commenced treatment, we believe it is fair to say that self-isolation based on a valid fear of transmitting disease is both a pragmatic approach to reducing TB transmission and a form of self-discrimination.

Patients' preference to hide their TB from other members of the community seems justified, since several community members reported that they did discriminate against people with TB. The multiple causes of this discrimination will require multiple interventions tailored to the local context and social system. The issue of perceived risk of infection that seems to be widespread throughout the community could be addressed by health education, but because this perception is closely associated with prejudice against the poor, people of low caste and people who engage in 'disreputable' behaviour, it may be very difficult to change this discriminatory behaviour, as has been observed with discrimination towards people with HIV/AIDS. (It should be noted in this context that in Nepal the prevalence of HIV is still very low, so that the association in people's minds between TB and HIV/AIDS is still low.)

It may be even more difficult to address perceptions that TB is in some way a curse sent as divine retribution for misdeeds of the person with TB or his family. It is likely that such religious beliefs will be difficult to change. It is not clear whether priests and other religious leaders in Nepal hold such beliefs, or make active efforts to refute them, or are passively accept them. Further research will be needed to clarify whether religious leaders are part of the solution or part of the problem, since this will influence the possibility of encouraging religious leaders to act as advocates for reducing discrimination associated with TB, or whether we need to first address discrimination amongst the leaders themselves. Further research is also needed to understand the processes by which incorrect information on TB is passed from generation to generation, which can then inform interventions to address misleading information that fuels discrimination.

Some patients felt they were discriminated against by health workers, and were able to quote specific examples of discrimination, generally at diagnosis. In our experience the vast majority of health workers understand, following repeated training, that they are at very little risk of being infected by TB patients after 2 weeks of treatment; but the extent of risks to health workers posed by TB suspects prior to diagnosis and by TB patients during the first two weeks of treatment are much less clear. It seems that there is still room for improvement in health-workers' behaviour towards TB patients. Perhaps 'education' is not enough – for if we tell health workers that patients are no longer infectious two weeks after starting treatment, the implication is that they are infectious for the first two weeks. Perhaps we really do need an intervention that protects health workers during those first two weeks. This may have two consequences: it will protect health workers; and it may raise their confidence that the health education messages they are receiving and passing on to patients are believable. Health workers can and should protect themselves: however, they should do so while behaving in a positive, supportive way to the symptomatic/patient, rather than presenting an unwelcoming negative attitude.

## Conclusion

In summary, basic population-wide health education is unlikely to be effective to address the multiple causes of discrimination identified: complex interventions will be needed. Further more detailed work is needed to understand the causes of discrimination in more depth, if we are to develop the well-informed interventions that are necessary to tackle this problem.

To end, we draw three conclusions about stigma and discrimination associated with communicable diseases more widely.

First, since discrimination and its links with stigma are very context-specific, only by performing studies in the specific setting where we want to develop interventions can we determine the key issues to be addressed: that is, whether we need to address the full range of discrimination, or whether we should or must narrow the focus to self-discrimination (as may be the case for TB in some settings), the stigmata themselves (as may be the case in leprosy) or the full range of stigma and its associated discrimination (as may be the case for HIV/AIDS).

Second, we reiterate our belief that the time has come to stop describing the discrimination due to communicable diseases, and concentrate our efforts on better understanding the causes of discrimination.

Finally, we make a plea for those involved in research, development and implementation of communicable disease control to acknowledge that the issue to be addressed is primarily discrimination, not stigma, and to give clear messages to that effect, rather than clouding the issue by referring to stigma when we mean discrimination.

## Competing interests

The author(s) declare that they have no competing interests.

## Authors' contributions

JN conceived of the study, participated in its design and coordination and drafted the manuscript. SB coordinated the study, participated in its design, performed the analysis and helped draft the manuscript. DK participated in the design of the study, performed the analysis and helped draft the manuscript.

## Pre-publication history

The pre-publication history for this paper can be accessed here:


